# The Cys-His-Gly triplet within the WNT motif is essential for Wnt16 function *in vivo*

**DOI:** 10.17912/micropub.biology.001736

**Published:** 2025-08-08

**Authors:** Emily G. Ramirez, Maria F. Rojas, Jyoti Rai, W. Joyce Tang, Claire J. Watson, Ronald Young Kwon

**Affiliations:** 1 Department of Orthopaedic Surgery and Sports Medicine, University of Washington, Seattle, Washington, United States; 2 Institute for Stem Cell & Regenerative Medicine, University of Washington, Seattle, Washington, United States

## Abstract

WNTs are critical to many developmental and disease processes. They are post-translationally acylated at a serine within a highly conserved sequence termed the "WNT motif". Changes in individual amino acids in the WNT motif reduce but do not eliminate WNT function. However, the role of a highly conserved triplet of residues (Cys-His-Gly) upstream of the serine has yet to be examined. We show that an in-frame deletion of the Cys-His-Gly triplet in zebrafish Wnt16 likely functions as a null mutation. These findings highlight the utility of using small in-frame indels that target conserved amino acid regions to modulate protein function.

**Figure 1. The Cys-His-Gly triplet within the WNT motif is necessary for Wnt16 function:**
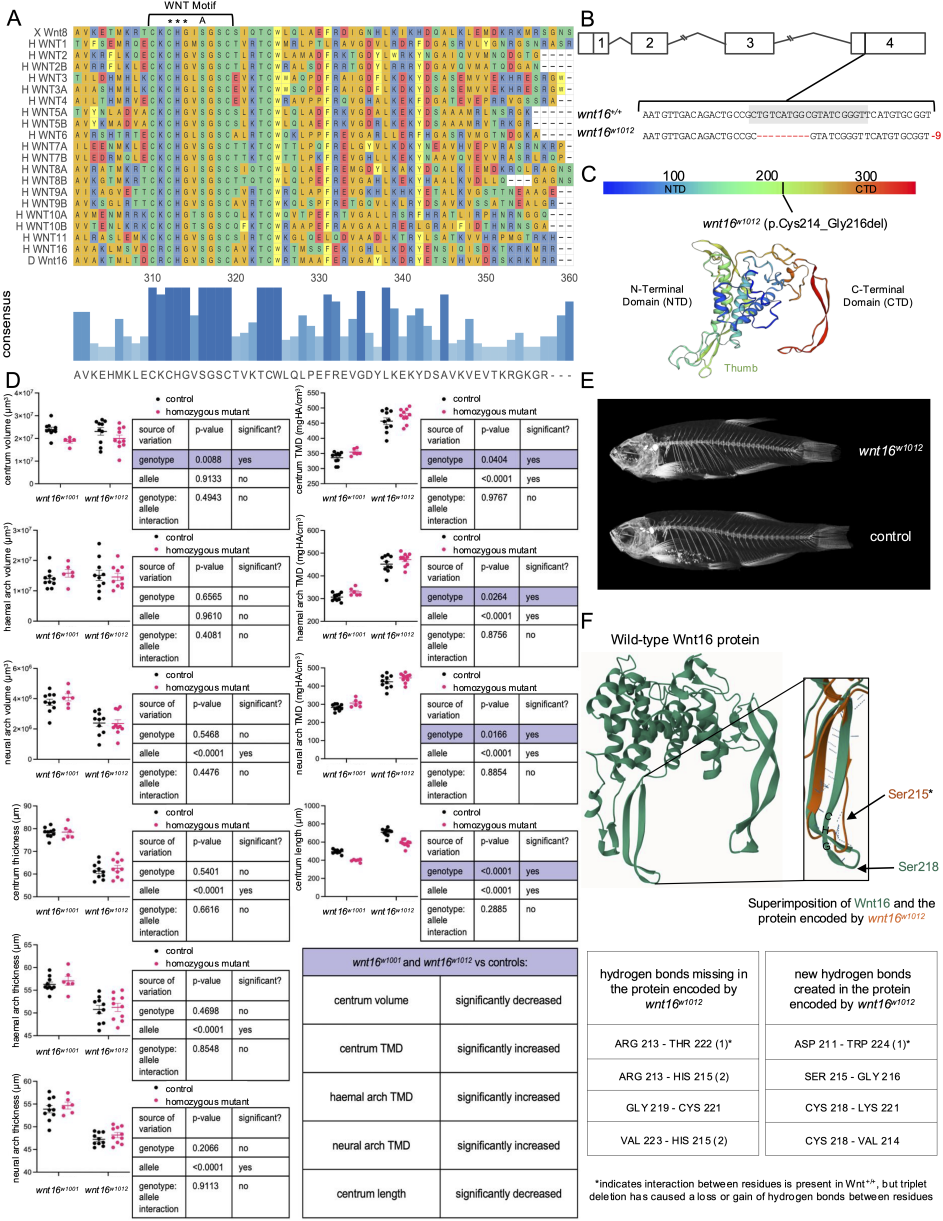
A. (Top) Sequence alignment of *Xenopus* (X) Wnt8, human (H) WNT1-16, and zebrafish (D) Wnt16. Asterisks indicate the highly conserved Cys-His-Gly triplet within the WNT motif. "A" indicates the serine that is post-translationally acylated. (Bottom) Darker and taller bars indicate higher consensus at that amino acid position. B. Sequence and genomic location of *wnt16 ^w1012^* . Grey highlight indicates gRNA target sequence in exon 4 used for CRISPR-based gene editing. C. Predicted effects of allele on amino acid sequence with location on protein indicated. D. 2-way ANOVA analyses of volume, tissue mineral density (TMD), and thickness for centrum, haemal arch, and neural arch, and centrum length. n=10/group for *wnt16 ^w1001^* clutchmate controls and *wnt16 ^w1012^* homozygous mutants and clutchmate controls. n=6/group for *wnt16 ^w1001^* homozygous mutants. Purple highlight and table indicate phenotypic features of *wnt16 ^w1001^* and *wnt16 ^w1012^* that differ significantly from controls. E. Maximum intensity projections of representative microCT scans for one *wnt16 ^w1012^* fish and one clutchmate control. F. (Top) Structural model of the wild-type Wnt16 protein (green) with inset showing superimposition of the protein encoded by *wnt16 ^w1012^* (orange). Blue dotted lines represent hydrogen bonds. “CHG” represents the location of the Cys-His-Gly triplet on Wnt16. Arrows indicate the location of acyl addition (*Ser215 is equivalent to Ser218 but shifted due to triplet deletion). (Bottom) Tables showing missing/created hydrogen bonds within the thumb of the encoded protein of *wnt16 ^w1012^* . Numbers in parentheses indicate the number of those specific bonds that are missing or created.

## Description

WNT proteins are highly conserved cysteine-rich glycoproteins critical to numerous developmental, regenerative, homeostatic, and pathological processes. Given their functional importance, it is essential to understand how specific mutations alter WNT protein function. While missense and loss-of-function variants for genes encoding WNT proteins have been well-characterized, much less is known about the functional consequences of small in-frame insertions and deletions (indels). Indels occur frequently within the genome and can have significant repercussions on protein structure (Savino et al., 2022; Miton and Tokuriki, 2023). Moreover, small in-frame indels have been linked to diseases involving WNT family members (Kantaputra et al., 2018). Therefore, there is a scientific need to better understand how small in-frame indels alter WNT protein function, as this could lead to principles that help to predict the consequences of genetic variation in WNT genes, as well as reveal novel strategies to manipulate WNT signaling.

Here, we examine the consequences of a small deletion in the so-called “WNT motif”, a sequence of highly conserved amino acid residues present in all 19 known human WNT proteins: (C-[KR]-C-H-G-[LIVMT]-S-G-x-C) (Laine et al., 2013) ( Fig. 1A ). The WNT motif resides in the N-terminal saposin-like “thumb” domain which has multiple functions including serving as the site of a post-translational acylation at a highly conserved serine, as well as facilitating the engagement of WNTs with Frizzled (FZD) receptors (Janda et al., 2012). Residues in the WNT motif have individually been found to play important roles in WNT function, predominantly through single amino acid substitutions. For example, Rios-Esteves et al. showed that substituting individual conserved amino acids within the WNT3A thumb domain limits—but does not eliminate—secretion and activity *in vitro* (Rios-Esteves et al., 2014). Although the effects of single amino acid substitutions in the WNT motif have been well-characterized, how small deletions in the WNT motif alter the function of WNT proteins remains unknown.

Using CRISPR/Cas9-based gene editing in zebrafish, we isolated the *wnt16 ^w1012^* mutant line (c.640_648delTGTCATGGC) ( Fig. 1B ). *wnt16 ^w1012^* is a 9 base-pair in-frame deletion resulting in loss of the Cys-His-Gly sequence within the WNT motif (p.Cys214_Gly216del) ( Fig. 1C ). WNT16 plays an important role in skeletal development, maintenance, and genetic influence on bone mineral density (Gómez et al., 2023). Recently, we showed that *wnt16* is necessary for spine and muscle morphology in zebrafish (Watson et al., 2022). In this study, we examined three different mutant alleles ( *w1001* , *w1008* , and *w1009* ) encoding for nonsense mutations predicted to result in the loss of key domains and residues critical for Wnt activity and secretion. Homozygous mutants for all three alleles phenocopied, indicating that all three alleles are likely functioning as null alleles (Watson et al., 2022). After the publication of Watson et al., we isolated the *wnt16 ^w1012^* mutant harboring a small in-frame deletion in the highly conserved WNT motif, offering a unique opportunity to evaluate the phenotypic consequences of loss of the Cys-His-Gly triplet in the WNT motif and how this compares to other *wnt16* mutants.

To investigate the phenotypic effects of the *wnt16 ^w1012^* mutation on adult vertebral morphology, we performed microCT scanning in *wnt16 ^w1012^* mutants and wild-type (WT) clutchmates at 90 days post fertilization (dpf). FishCuT, a segmentation algorithm developed by our lab for microCT-based phenotyping in the zebrafish skeleton, was used to measure volume, tissue mineral density (TMD), thickness, and centrum length for the centrum, haemal arch, and neural arch of the 20 anterior-most pre-caudal and caudal vertebrae, resulting in 200 measures per fish (Hur et al., 2017). FishCuT analysis revealed significant differences between WT fish and *wnt16 ^w1012^* mutants: *wnt16 ^w1012^* mutants (n=10/group) exhibited significantly reduced centrum lengths (p<0.0001), along with a significant increase in neural arch angles (p=0.0002) ( Fig. 1E ). Thus, adult *wnt16 ^w1012^* mutants exhibit altered spine morphology.

In order to better understand the impacts of loss of the Cys-His-Gly on Wnt16 function, we compared *wnt16 ^w1012^* mutant phenotypes to those of *wnt16 ^w1001^* mutants, which our previous studies indicated was functioning as a null allele (Watson et al., 2022). We used two-way ANOVA statistical tests to formally compare *wnt16 ^w1012^* and *wnt16 ^w1001^* mutant phenotypes. For this, we used the average of each of the 10 FishCuT measurements. The two-way ANOVA allowed us to simultaneously determine whether: (1) there were common phenotypic differences between WT and mutants for both alleles (indicated by *p* -value for genotype), (2) there were baseline phenotypic differences for different alleles due to genetic background and/or environmental conditions when testing each allele (indicated by *p* -value for allele), and (3) mutants for each *wnt16* allele exhibited different phenotypic responses when compared to WT (indicated by *p* -value for genotype:allele interaction). Two-way ANOVA analyses confirmed significant effects of genotype for centrum length (p<0.0001) and revealed significant effects of genotype for centrum volume (p=0.0088) and centrum (p=0.0404), haemal arch (p=0.0264), and neural arch (p=0.0166) TMD ( Fig. 1D, E). While we observed significant effects of allele impacting both controls and mutants for most measures ( Fig. 1D ), this is unlikely to be due to differences in genetic background, as both *wnt16 ^w1012^* and *wnt16 ^w1001^* mutants were generated in an AB background but tested at different times. Rather, this is likely due to slight changes in the environment when testing each allele and their clutchmate controls, which resulted in different developmental rates. Notably, there were no significant genotype:allele interactions across any of the ten measurements analyzed ( Fig. 1D ). Therefore, the phenotypic responses in *wnt16 ^w1012^* and *wnt16 ^w1001^* mutants were similar, suggesting that *wnt16 ^w1012^* is functioning similarly to a null allele.

We next sought to determine the structural impact of the Cys-His-Gly triplet deletion on the Wnt16 protein. Structural modeling of the encoded protein of *wnt16 ^w1012^* and superimposition with WT Wnt16 suggested conformational changes within the thumb domain resulting from the triplet deletion ( Fig. 1F ) (Confidence: 0.70) (Baek et al., 2021). Specifically, the predicted protein encoded by *wnt16 ^w1012^* exhibited alteration of multiple hydrogen bonds and truncation of the antiparallel beta sheets within the thumb domain. Notably, several native hydrogen bonds in WT Wnt16 were lost, while novel, aberrant hydrogen bonds were formed in the mutant ( Fig. 1F ). Loss of the Cys within this motif additionally resulted in the loss of the native disulfide bond involving this residue. These changes suggest that deletion of the conserved Cys-His-Gly triplet may significantly alter WNT structure and potentially, its function.

Taken together, our *in vivo* studies and structural modeling indicate that the Cys-His-Gly triplet within the WNT motif is essential for Wnt16 function. Given the highly conserved nature of the WNT motif, it is likely that this function is shared amongst all WNT family members harboring this sequence. Prior studies of genetic variation in WNTs have almost exclusively focused on nonsense mutations or missense mutations. These studies have shown that single amino acid substitutions in Wnt proteins reduce but do not eliminate secretion and/or activity (MacDonald et al., 2014; Rios-Esteves et al., 2014). Conversely, while prior studies have shown the potential for nonsense mutations in WNT genes to be deleterious, premature termination codons could trigger genetic compensation by mutant mRNA degradation (El-Brolosy et al., 2019). Moreover, truncated WNT protein products could have residual or even dominant negative effects (Hoppler et al., 1996). Our findings suggest that a small in-frame deletion of the Cys-His-Gly triplet in the WNT motif eliminates WNT function. A limitation of this study is that it is unknown whether Wnt16 secretion or activity is affected by the loss of the Cys-His-Gly triplet. However, this could be investigated in future studies with the development of suitable Wnt16-specific antibodies. The results we report here contribute to our understanding of the consequences of small in-frame deletions in the WNT motif and support the utility of using small in-frame indels that target conserved amino acid regions to modulate protein function.

## Methods

Ethics statement:

These experiments were conducted on an approved protocol in accordance with the University of Washington Institutional Animal Care and Use Committee (IACUC).

Zebrafish care and genotyping:

Zebrafish used in these experiments were housed in a facility on a 14:10 hour light:dark photoperiod. All fish were housed in plastic tanks at 28.5 degrees C on a commercial recirculating aquaculture system and fed a commercial diet. Conducted studies used mixed sex WT (AB) and mutant ( *wnt16 ^w1012^* ) lines. Clutches of the heterozygous and homozygous mutants were housed together. Experimental animals were generated by incrossing heterozygous *wnt16 ^w1012^* zebrafish.

Genotyping was performed by taking fin clip samples from adult (90 days post fertilization (dpf)) zebrafish and using standard PCR procedures (35 cycles, 58°C annealing temperature). The following primers were used for *wnt16 ^w1012^* (F: 5’- CATGCTCTCCGTGTCCTGTT-3’, R: 5’- ATCCTTGCGTCGCACCTTAC-3’). Gel electrophoresis on 3% high-resolution agarose gels identified WT and mutant alleles based on amplicon sizes.

CRISPR/Cas9-based gene editing:

CRISPR mutagenesis was performed using the Alt-R CRISPR-Cas9 System from Integrated DNA Technologies (IDT). The gRNA duplex was generated by mixing tracrRNA with crRNA in a 1:1 ratio. This mixture was then incubated at 95 degrees C for 5 minutes and cooled on ice. Ribonucleoprotein complexes were made by mixing Alt-R S.p. HiFi Cas9 Nuclease with a 3XNLS sequence with the crRNA:tracrRNA gRNA duplex in a 1:1 ratio. This mixture was then incubated at room temperature for 5-10 minutes, producing the Cas9:gRNA RNP complex at ~25 mM for injection. Pre-pulled microcapillary needles (Tritech Research) were loaded with RNPs and calibrated. Yolks of 1- to 4-cell stage zebrafish embryos were injected with 2 nL RNP complexes. The following crRNA guide target sequence was used to target exon 4 of *wnt16* : (5’—AACCCGATACGCCATGACAG—3’).

Micro-computed tomography (microCT) scanning and analysis: Fish were imaged at 90 dpf using a Scanco vivaCT 40 microCT scanner. Acquired scans with 21 μm voxel size were taken using these settings: 500proj/180 °, 145mA, 1024 samples, 55kVp, 200 ms integration time. Scanco software generated DICOM files of each fish, which were then used for maximum intensity projections of DICOM images. Scans of 4 fish were taken simultaneously in each acquisition. Maximum intensity projections were used for analysis.

FishCuT was used to segment the vertebrae, and analysis was performed as previously described (Hur et al., 2017).

Statistical analysis:

The majority of results are from a single experiment. One technical replicate is represented by each biological replicate. Empirical data are represented as mean ± SEM or as individual measurements. Figure caption reports group sizes (n). No outliers were identified, and all data were included in statistical analyses. The global test using the globaltest package in R was used to perform multivariate analysis of vertebral data. GraphPad Prism was used to conduct all other statistical tests. p<0.05 was considered statistically significant across all data.

Structural modeling:

PDB files of our proteins of interest were generated using the RoseTTAFold method through Robetta (Baek et al., 2021). Computational models were created using Mol* Viewer (Sehnal et al., 2021). PDB files were uploaded, and regions of interest, with hydrogen bonds showing, were superimposed. Hydrogen bond analysis involved manual comparison of bonds between the superimposed regions.

## Reagents

**Table:** 

**Reagent or Resource**	**Source**	**Identifier**
*Genotyping*		
DreamTaq DNA Polymerase	Thermo Fisher Scientific	Cat# EP0711
Proteinase K	Thermo Fisher Scientific	Cat# EO0491
		
*Sequencing*		
GeneJET Gel Extraction Kit	Thermo Fisher Scientific	Cat# K0692
		
*Primers*		
* wnt16 ^w1012^* genotyping primers: Fwd, CATGCTCTCCGTGTCCTGTT; Rev, ATCCTTGCGTCGCACCTTAC	This paper	
		
*CRISPR Cas9*		
Alt-R CRISPR-Cas9 System	Integrated DNA Technologies	Cat# 1072532
* wnt16 ^w1012^* gRNA: AACCCGATACGCCATGACAG	This paper	
		
*Zebrafish Lines*		
* wnt16 ^w1001^*	Watson et al. 2022	ZIRC Catalog ID: ZL14731
* wnt16 ^w1012^*	This paper	ZIRC Catalog ID: ZL14818
		
*Software*		
GraphPad Prism (Prism 9.5.0)	Graph Pad Software	https://www.graphpad.com/
Fiji (ImageJ 2.16.0)	Schindelin et al. 2012	https://fiji.sc/
FishCuT (FishCuT 1.2)	Hur et al. 2017	
RoseTTAFold (Robetta)	Baek et al., 2021	https://robetta.bakerlab.org/
Mol* Viewer (Mol* Plugin 3.40.1)	Sehnal et al., 2021	https://molstar.org/viewer/
